# Quantitative analysis of therapeutic proteins in biological fluids: recent advancement in analytical techniques

**DOI:** 10.1080/10717544.2023.2183816

**Published:** 2023-03-06

**Authors:** Jae Geun Song, Kshitis Chandra Baral, Gyu-Lin Kim, Ji-Won Park, Soo-Hwa Seo, Da-Hyun Kim, Dong Hoon Jung, Nonye Linda Ifekpolugo, Hyo-Kyung Han

**Affiliations:** BK21 FOUR Team and Integrated Research Institute for Drug Development, College of Pharmacy, Dongguk University-Seoul, Goyang, Korea

**Keywords:** Protein drugs, protein assays, LC-MS/MS, ELISA, chromatographic separation, quantitation

## Abstract

Pharmaceutical application of therapeutic proteins has been continuously expanded for the treatment of various diseases. Efficient and reliable bioanalytical methods are essential to expedite the identification and successful clinical development of therapeutic proteins. In particular, selective quantitative assays in a high-throughput format are critical for the pharmacokinetic and pharmacodynamic evaluation of protein drugs and to meet the regulatory requirements for new drug approval. However, the inherent complexity of proteins and many interfering substances presented in biological matrices have a great impact on the specificity, sensitivity, accuracy, and robustness of analytical assays, thereby hindering the quantification of proteins. To overcome these issues, various protein assays and sample preparation methods are currently available in a medium- or high-throughput format. While there is no standard or universal approach suitable for all circumstances, a liquid chromatography-tandem mass spectrometry (LC-MS/MS) assay often becomes a method of choice for the identification and quantitative analysis of therapeutic proteins in complex biological samples, owing to its high sensitivity, specificity, and throughput. Accordingly, its application as an essential analytical tool is continuously expanded in pharmaceutical R&D processes. Proper sample preparation is also important since clean samples can minimize the interference from co-existing substances and improve the specificity and sensitivity of LC-MS/MS assays. A combination of different methods can be utilized to improve bioanalytical performance and ensure more accurate quantification. This review provides an overview of various protein assays and sample preparation methods, with particular emphasis on quantitative protein analysis by LC-MS/MS.

## 1. Introduction

Proteins-based therapeutics display diverse functions and high target selectivity in pathological conditions (Zheng et al., 2014a). Therefore, protein drugs are actively pursued for the treatment of various diseases, and the global pharmaceutical market for protein drugs is continuously expanding with new drug approvals (van den Broek et al., 2013). To expedite the identification and preclinical/clinical development of new protein drugs, sensitive and reproducible protein assays and analytical tools are essential to the pharmaceutical R&D process (van den Broek et al., 2013). In particular, a reliable quantitative assay for protein drugs in biological fluids such as plasma and urine can be instrumental in determining the pharmacokinetic profiles of therapeutic proteins (Russell et al., 2020, 2021). To date, diverse analytical assays are available for quantifying protein content in *in vitro* and *in vivo* samples, including UV absorption methods, colorimetric assays, immunoassays, and chromatographic analyses (Chang & Zhang, 2017; Kim et al., 2022a). These methods have their own advantages and disadvantages in terms of assay time, sensitivity, compatibility, specificity, and robustness. Of note, the severe interference from endogenous proteins and substances is often a big challenge in quantifying protein drug concentrations in biological fluids (Gillette & Carr, 2013). Analytical methods should be selected depending on the accuracy, sensitivity, and specificity required for determining protein concentration.

Recently, liquid chromatography-tandem mass spectrometry (LC–MS/MS) has been widely adopted as a method of choice for the quantitative analysis of protein drugs in biological fluids owing to its high specificity and sensitivity (Plumb et al., 2012). The LC–MS/MS assay is moderately high-throughput, achieving high sensitivity and specificity for target proteins in complex biological matrices (Plumb et al., 2012). However, the optimization of LC-MS/MS conditions is not straightforward, and there are many factors to be considered in sample preparation, chromatographic separation, and optimizing mass spectrometry conditions. Despite these complications in optimizing LC-MS/MS conditions in regard to sensitivity, accuracy, and specificity, advances in instrument and analytical techniques have promoted LC-MS/MS as a more practical assay for monitoring therapeutic proteins and their potential metabolites in plasma during clinical development. In general, there are two approaches for protein quantitation by LC-MS/MS: bottom-up approach and top-down approach. Bottom-up approach involves the enzymatic protein digestion into smaller peptides and then one or more unique signature peptides are separated and analyzed by LC-MS/MS (Kang et al., 2020). On the other hand, top-down approach employs the direct measurement of intact protein by LC-MS/MS. While bottom-up approach is relatively fast and easy to implement, top-down approach shows the improved selectivity, enabling to characterize the hard-to-predict events such as coding polymorphisms, alternative splicing, and posttranslational modifications, simultaneously (Patrie & Cline, 2020). Nowadays, top-down approach is widely used for protein quantitation by LC-MS/MS.

Given that the currently available protein assays have their own advantages and disadvantages, there is no single universal method suitable for all proteins; method selection should be based on the compatibility with the samples to be analyzed. For the best choice of an analytical assay, it is important to understand the working mechanisms, assumptions, and limitations of each analytical method. Therefore, this review will provide a brief overview of protein assays applicable for the quantification of protein drug concentration, with a focus on LC-MS/MS assays as recent advances in LC-MS/MS expand the applicability of this technique during the preclinical and clinical development of protein drugs.

## 2. Quantitative analytical methods for protein-based drugs

### 2.1. Ultraviolet (UV) absorption method

Proteins that contain aromatic amino acids such as tyrosine, tryptophan, and phenylalanine, exhibit strong UV absorption around 230–300 nm (Antosiewicz & Shugar, 2016; Yanti et al., 2021). In addition, the peptide bond in proteins has a strong absorption around 190 nm and a weak absorption between 210 and 220 nm (Prasad et al., 2017). Recent studies also suggest that proteins rich in charged amino acids (e.g. Lys, Glu, Arg) can display distinct UV absorption at 250–350 nm although they lack aromatic amino acids (Prasad et al., 2017). As UV absorption is proportional to chromophore content and total protein concentration, the measurement of UV absorption at specific wavelengths can be a simple and direct assay method for determining protein concentrations based on Beer-Lambert law. The measurement of UV absorption does not require any assay reagents. It is rapid, cost-effective, high-throughput, and does not affect the biological activity of proteins (Aitken & Learmonth, 2009; Kielkopf et al., 2020). Furthermore, it does not affect the biological activity of proteins. However, the UV absorption method is not applicable for protein mixtures and for samples containing non-protein contents that can absorb UV light. For example, nucleic acids have strong UV absorbance at 280 nm due to which the coexistence of nucleic acids will interfere with the protein assay by UV absorption (Kielkopf et al., 2020). Interference can also be caused by solvents and various components of biological buffers (Noble & Bailey, 2009; Reinmuth-Selzle et al., 2022). Therefore, interference from co-existing components in biological matrices should be carefully examined during sample preparation and the selection of optimal wavelength.

### 2.2. Colorimetric methods

#### 2.2.1. Bradford assay

The colorimetric reagent Coomassie Brilliant Blue G −250 dye (triphenylmethane dye) can be used to measure the total protein concentration in a sample as described by Bradford in 1976 (Bradford, 1976; Kielkopf et al., 2020). Although the precise mechanism of the Bradford assay is not fully defined, the main mechanism is the binding of Coomassie dye to basic amino acid residues (e.g. arginine, lysine, and histidine) under acidic conditions, leading to a color change from brown to blue (He, 2011; Reinmuth-Selzle et al., 2022). The protein-dye complex stabilizes the anionic form of the dye, resulting in a spectral shift from the reddish-brown form of the dye (maximum absorbance at 465 nm) to the blue form (maximum absorbance at 610 nm) (Olson, 2016; Filgueiras & Borges, 2022). Although the optimal wavelength is 595 nm, the amount of blue color (absorbance) can be measured at 575–615 nm if needed (Karimi et al., 2022).

Among amino acid residues, arginine residues mainly interact with the Coomassie dye while other basic amino acids (lysine and histidine) only give slight responses (Reinmuth-Selzle et al., 2022). This may explain the wide protein-to protein variation in Bradford assays. Van der Waals forces and hydrophobic interactions may also influence protein binding to the dye (Reinmuth-Selzle et al., 2022).

The Bradford assay is simple, economic, and fast (Reinmuth-Selzle et al., 2022). It is a sensitive technique that can detect a large range of proteins. In addition, it is compatible with buffers, solvents, thiols, metal ions, reducing substances, and chelating agents (Krohn, 2005). The Bradford assay can be performed in a test tube or in microplate format that may serve as a high-throughput assay. However, it also possesses some disadvantages. The Bradford assay is not suitable for quantifying low-molecular weight peptides or proteins (less than 3,000 Da) that do not produce color by binding with the Coomassie dye (Cohen & Walt, 2019). Nor is it applicable to proteins that are poorly soluble under acidic conditions (Olson, 2016). The assay is incompatible with various ionic and nonionic surfactants (e.g. SDS, Triton X), causing precipitation of the Coomassie dye reagent (Olson, 2016). Recently, modified Bradford assay kits are commercially available and show improved compatibility with certain surfactants and increased linearity of response (Scientific, 2013; Zhou et al., 2022).

#### 2.2.2. Lowry assay

As described first by Lowry in 1951, copper-based protein assays depend on the biuret reaction as a first step. In the biuret reaction, proteins form a colored chelate complex with cupric ions (Cu^2+^) in the presence of sodium potassium tartrate. Biuret reacts with copper to form a light blue, tetradentate complex (Martina & Vojtech, 2015). Following the reduction of cupric cations (Cu^2+)^ to cuprous cations (Cu^+^), the addition of the Folin-Ciocalteu reagent (phosphomolybdate-phosphotungstate) enhances the light blue color of the tetradentate copper-protein complex (maximum absorbance at 750 nm), increasing the sensitivity of the biuret reaction (Noble & Bailey, 2009; Goldring, 2012).

Compared to the Bradford assay, the Lowry assay is compatible with most surfactants and exhibits less protein–protein variation (Hayes, 2020). It is more sensitive than the UV absorption method (Rodger & Sanders, 2017). However, this method requires more time and reagents than the Bradford assay. In addition, a high proline content will interfere with the protein-copper complex formation (Schaich, 2016). Since color development is not only due to the reduced copper-amide bond complex but also due to amino acid residues such as tyrosine and tryptophan (or cystine, cysteine, and histidine to a lesser extent), this assay exhibits variability according to protein sequence (Le et al., 2016; Schaich, 2016). Samples containing ammonium sulfate, free thiols, sucrose, chelating agents (EDTA), Tris-HCl, strong acids or strong bases, and Triton X could also compromise the sensitivity of this protein assay (Schaich, 2016). Consequently, modified Lowry methods have been proposed to use fewer reagents, increase the speed and stability of the color formation, improve the compatibility with some salt solutions, and provide a more linear response (Olson, 2016; Schaich, 2016). There are also many commercial sources of the modified Lowry assay.

#### 2.2.3. Bicinchroninic acid (BCA) assay

The BCA assay is a widely used method for the quantitation of total proteins in solution (Ahlschwede et al., 2022; Tran & Park, 2022). Similar to the Lowry assay, the BCA assay is a copper-based protein assay where Folin-Ciocalteu reagent in the Lowry assay is replaced by BCA. This method is based on two steps—protein-copper chelation and the secondary detection of the reduced copper (Walker, 2009). The first step is the biuret reaction, where proteins produce the blue chelate complex by reacting with cupric ions (Walker, 2009). Following the reduction of cupric cations (Cu^2+^) to cuprous cations (Cu^+^), the reduced cuprous cations (Cu^+^) react with a highly sensitive and selective colorimetric detection reagent, BCA, resulting in an intense purple BCA/copper complex (λ_max_ = 562 nm) (Olson, 2016). The BCA/copper complex is water-soluble and its absorbance at 562 nm exhibits strong linearity with increasing protein concentrations. In addition to 562 nm, the purple color can be measured at any wavelength of 550 nm–570 nm with minimal (<10%) loss of signal.

The BCA assay has many advantages over other protein assays. Compared to the Bradford assay, it is more sensitive and compatible with surfactants at concentrations up to 5%. In addition, it responds more uniformly to different proteins than the Bradford method, exhibiting lower protein-protein variation (Noble et al., 2007). In the BCA assay, the reagent is fairly stable under alkaline conditions and can be included in the copper solution, leading to a one-step process. It is applicable over a broad range of protein concentrations (0.5 μg/mL to 1.5 mg/mL). However, it also displays some disadvantages. Substances reducing or chelating copper will produce color, thus interfering with the accuracy of the protein quantitation (Noble et al., 2007). The accuracy of the results is also affected by the presence of acidifiers, reducing sugars, lipids, and phospholipids in the buffer. Variable colorimetric responses may arise due to differences in the compositions and structures of proteins, including amino acid sequence, isoelectric point, secondary structure, and side chain or prosthetic groups (Krohn, 2005). The intensity of the produced color also varies with the incubation time and temperature, requiring the optimization of assay conditions for each application (Krohn, 2005). At present, different BCA assay kits, including the micro-BCA protein assay, are commercially available.

### 2.3. Fluorescence-based assay

Fluorescence-based assays for protein quantitation have been developed to overcome the limitations of absorbance-based assays. In particular, fluorescence-based protein assays may be a better option for total protein quantitation where sensitivity or limited sample size might be an issue. These assays have superior sensitivity and lower background signals, requiring less protein sample for quantitation than colorimetric protein assays (An, 2009). They are suitable for high-throughput application with automation to quantify proteins in biological samples. Furthermore, they provide a wider dynamic range and lower protein-to-protein variability than colorimetric assays (An, 2009).

Fluorescence-based assays detect the fluorescent signal from the dye attached to proteins, using a fluorometer or a microplate reader. In general, fluorescence-based protein assays adopt two different approaches: (i) using nonfluorescent but reactive dyes that covalently bind to the amine groups of proteins and form fluorescent adducts, or (ii) using dyes that exhibit fluorescence enhancement upon non-covalent interaction with hydrophobic regions of proteins or detergent-coated proteins (Jones et al., 2003). For both approaches, various fluorescence probes have been developed and are commercially available. For example, fluorescamine is nonfluorescent but interacts with primary amines, producing fluorescent adducts detectable at λ_ex_ = 390 nm and λ_em_ = 475 nm (Jones et al., 2003). Similarly, *o*-phthaldialdehyde (OPA) and 3-(4-carboxybenzoyl) quinoline-2-carboxaldehyde (CBQCA) are nonfluorescent but react with primary amine groups of proteins (Bantan-Polak et al., 2001). CBQCA binds to primary aliphatic amines in the presence of cyanide or thiols, leading to the formation of a fluorescent adduct detectable at λ_ex_ = 450 nm and λ_em_ = 550 nm (You et al., 1997; Bantan-Polak et al., 2001). OPA is also nonfluorescent but reacts with primary amines in the presence of 2-mercaptoethanol, producing an intense blue, fluorescent product detectable at λ_ex_ = 340 nm and λ_em_ = 455 nm (Bantan-Polak et al., 2001; Jones et al., 2003). These reagents function well in the presence of lipids that normally interfere with protein estimations (Olson, 2016). They are also compatible with reducing agents, metal chelators, and detergents (Jones et al., 2003). However, they are not compatible with amine-containing buffers (e.g. Tris or glycine buffers), and the presence of free amino acids or other materials containing primary amines interferes with protein detection (Jones et al., 2003). The sensitivity of their assays depends on the number of amines in the protein analytes. In addition to these reagents, coating proteins with detergent and detecting detergent-protein complexes are also known to improve the detection and quantitation of proteins, since certain ionic detergents can coat proteins with near uniformity (Jones et al., 2003). At present, nonfluorescent dyes that become intensely fluorescent upon binding to detergent-coated proteins or hydrophobic regions of proteins are commercially available for protein quantitation in complex biological samples.

### 2.4. Immunoassays

The immunoassays are highly selective bioanalytical methods for the detection or quantitation of diverse biomacromolecules (Darwish, 2006). Owing to their high specificity and sensitivity, immunoassays can be used to quantify very low concentrations of proteins in biological samples. In addition, they can be used to analyze large numbers of samples at a time without exhaustive sample preparation, thus minimizing the turn-around time of analysis (Hsieh & Rao, 2017). Consequently, immunoassays have been widely used in the pharmaceutical development process of protein drugs for pharmacokinetic evaluation, bioequivalence studies, and drug monitoring. However, immunoassays for quantifying proteins in biological fluids may have a limitation linked to the interference from certain substances that may cause either false-negative or false-positive results. They often suffer from a lack of specificity and cross-reactivity with other molecules, including metabolites. In addition, suitable antibodies may not always be available.

To improve the sensitivity, specificity, and throughput, there has been continuous effort to refine immunoassays along with the development of new reagents, systems, and methodologies. Monoclonal antibodies are now widely used in immunoassays, and the methodologies of labels and solid-phase components are much more sophisticated. Given that the major trend is to avoid radioisotopic labels and move toward fast, reliable, and automated solid-phase assays with nonisotopic labels, some immunoassays with nonisotopic labels will be covered in the following section.

#### 2.4.1. Enzyme-linked immunosorbent assay (ELISA)

Enzymatic immunoassays are categorized into homogeneous enzymatic immunoassays and heterogeneous enzymatic immunoassays. In homogeneous enzymatic immunoassays, enzymes are inactivated while they bind to the antibody, eliminating the need for the washing step (Porstmann & Kiessig, 1992). They are easy to use but their application is limited due to high cost and low sensitivity. Compared to homogeneous enzymatic immunoassays, heterogeneous enzymatic immunoassays require washing steps to remove free, unbound antigen (Aydin, 2015) but are more commonly used due to their higher sensitivity (Aydin, 2015). In 1971, Engvall and Perlmann reported a simple, rapid, sensitive, and high-throughput, heterogeneous immunoassay technique, generally known as the enzyme immunoassay (EIA) or the enzyme-linked immunosorbent assay (ELISA) (Engvall & Perlmann, 1971). ELISA is a powerful method that is used to detect and quantitate very low concentrations of proteins, peptides, antibodies, and hormones in complex biological fluids with minimal interference. In principle, ELISA is a plate-based assay technique, where the antigen (target macromolecule) is immobilized on a solid surface and then complexed with a reporter enzyme-linked antibody (Hosseini et al., 2018). Detection occurs by measuring the activity of the reporter enzyme via incubation with the appropriate substrate to produce a measurable product. In this assay, horseradish peroxidase (HRP) and alkaline phosphatase (AP) are the most commonly used enzyme labels, although other enzymes, including β-galactosidase, catalase, glucose oxidase, and acetylcholinesterase, have also been used (Porstmann & Kiessig, 1992). In addition, various substrates are commercially available for ELISA. Substrates should be selected depending on the required assay sensitivity and the instrumentation for signal detection (e.g. spectrophotometer, luminometer, or fluorometer). For example, HRP conjugates with 5-amino salicylic acid and orthophenylenediamine produce brown complexes, while AP conjugates with sodium azide produce yellow complexes (Aydin, 2015). These enzyme-substrate reactions are usually completed within 30–60 min, and the absorbance is determined using a spectrophotometer at 400–600 nm depending on the characteristics of the conjugate used (Aydin, 2015).

Several different ELISA formats have been developed to increase the specificity of measurement. Among them, the sandwich ELISA assay is the most widely used due to its high sensitivity and specificity. In the sandwich ELISA, the analyte to be measured is bound between two primary antibodies—the capture antibody and the detection antibody—that detect different epitopes of the antigen (Drijvers et al., 2017). Initially, the sample is added to the microplate wells coated with the antibody. Then, the plate is incubated and undergoes washing steps to remove unbound antigens. Following the washing step, antibodies that are specific to the antigen are added and incubated; these antibodies are tagged with the reporter enzyme. The enzyme substrate is then added to the medium for producing colored complexes. Currently, matched pairs are commonly used in the sandwich ELISA where the capture and the detection antibodies do not interfere with each another and have simultaneous binding capacity.

ELISA is easy to perform due to the binding and immobilization of reagents to the solid surface. The immobilization of reactants to the microplate surface facilitates the separation of bound materials from unbound ones. Furthermore, the use of high-affinity antibodies and the washing away of nonspecific bound materials make ELISA a powerful tool for measuring specific analytes in a biological matrix. Furthermore, various ready-to-use ELISA kits are commercially available, allowing easy analysis of protein samples. Consequently, the ELISA is often a method of choice for quantifying protein concentrations in biological fluids owing to its speed, simplicity, specificity, and relatively low cost, even when proteins can be measured by other standard procedures. However, it also presents some drawbacks, including high cost, laborious assay procedures, antibody instability, insufficient blocking of immobilized antigens, and the possibility of cross-reaction (Shah & Maghsoudlou, 2016; Hosseini et al., 2018).

#### 2.4.2. Fluoroimmunoassay

Given that the detection step largely determines the sensitivity of immunoassays, the detection of antibodies can be done using fluorescent probes in addition to the use of immunoassays based on color intensity. Such assays are known as fluorescent immunoassays (FIAs) (Rizzo, 2022). The FIA is a variant of the ELISA, where the substrate used does not generate color but emits fluorescence. In addition to conventional FIAs, advancement in fluorescent labeling technologies and instruments has facilitated the development of various FIA-related methodologies, including fluorescence polarization immunoassays and time-resolved fluorescence immunoassays. Although standard fluorometric detection with conventional fluorophores is popular, it has several limitations: (i) a high background signal due to the simultaneous excitation/emission process, (ii) self-quenching due to relatively small Stokes shift (the difference between the maximum absorbance and emission wavelengths), (iii) a background signal from autofluorescent substances in biological matrices, and (iv) potential false positives due to fluorescent test compounds in high-throughput screening formats (Soini & Hemmilä, 1979; Hemmilä, 1985). In particular, background fluorescence from the sample can limit the utility of FIAs. To overcome these drawbacks, time-resolved fluoroimmunoassay techniques (TRFIAs) use chelates of lanthanides as fluorescent labels (Hagan & Zuchner, 2011). During standard fluorometric detection, the light emitted by the sample is measured while excitation occurs. However, in TRFIAs, lanthanide chelate labels allow the detection of the emitted light after excitation has occurred. Lanthanide chelates (e.g. europium, terbium, and samarium) have longer fluorescence lifetimes (µseconds–milliseconds) than the typical background fluorescence of biological matrices, enabling the measurement of fluorescence emission after any background fluorescence has decayed (Kricka & Park, 2014). In addition, lanthanide chelates have substantial Stokes shift, thus greatly increasing the signal to background ratio. Other unique characteristics of lanthanide chelates include the possibility of dissociating the label into a new, highly fluorescent chelate by pH shift and narrow emission peaks, which make the TRFIA a highly sensitive method associated with lower interference detection (Hagan & Zuchner, 2011; Rizzo, 2022).

The fluorescence polarization immunoassay (FPIA) is also a widely used homogeneous FIA. The polarization of the fluorescence from a fluorescence-labeled antigen (tracer) is determined by its rate of rotation during the lifetime of the excited state in solution. Since the degree of depolarization depends on the size of the molecule (large molecules rotate more slowly), a fluorescence-labeled tracer in solution has a lower degree of polarization than an antibody-bound tracer having slower rotation (Hendrickson et al., 2020). Therefore, fluorescence polarization can be modulated by the competition between drugs in the sample and the fluorescence-labeled tracer for binding to the antibody, where the depolarization is related to the drug concentrations in the sample (He et al., 2018). At low drug concentrations, more tracer molecules are bound to the antibody, leading to a higher fluorescence polarization.

Overall, the FIA is more sensitive than the ELISA and is used to determine low concentrations of proteins. It is also simple, easy to run, and rapid. The FIA is advantageous over the chemiluminescence immunoassay in terms of low cost, large signal intensity, and faster imaging speed (Ahmed et al., 2020).

#### 2.4.3. Chemiluminescence immunoassay

The basic principle of chemiluminescence immunoassays (CLIA) is similar to that of the ELISA, except that the substrate of the CLIA generates a luminescence signal in the presence of an enzyme, instead of developing a particular color. Chemiluminescence is the emission of light by a chemical reaction, where the enzyme used in CLIA converts the substrate to a reaction product emitting a photon of light (Rizzo, 2022). These chemiluminescent reactions can also be elevated by an enhancer that boosts the electronic activation and provides an intense light emission for a longer period, leading to highly enhanced analytic sensitivity (Cinquanta et al., 2017). Compared to ELISA, the CLIA shows higher sensitivity and shorter turnaround times. In addition, it has a wider dynamic range with a linear relationship between luminous intensity and the concentration of the measured substance. The key advantages of CLIA also include the absence of interfering emissions (high specificity), rapid acquisition of the analytical signal, and high stability of reagents and their conjugates.

Different types of substrates are available for CLIAs, and activation of these substrates requires chemical or enzymatic reactions associated with the immunological reactions (Cinquanta et al., 2017). Most chemiluminescent substrates are HRP-dependent, although AP-based substrates are also available in certain cases (Rizzo, 2022). Luminol or its derivatives are the most commonly used substrates in the presence of HRP and a peroxide buffer (Rizzo, 2022). The electro-chemiluminescence immunoassay (ECLIA) is another type of a CLIA, using electrical current for oxidizing substrates. The ECLIA also has higher sensitivity and a shorter analysis time than the ELISA (Bolton et al., 2020). Technical advancement will continuously encourage the development of a new and automated analytical method based on the principle of CLIA, increasing the assay efficiency and steadily reducing cost.

### 2.5. Chromatographic analysis

#### 2.5.1. High-performance liquid chromatography (HPLC)

In recent decades, HPLC has become an indispensable method for the purification, separation, and quantitation of peptides and proteins, owing to its high resolution, reproducibility, selectivity, and high recovery (Aguilar, 2004). There are various separation modes of HPLC, including ion exchange chromatography (IEC), size exclusion chromatography (SEC), reversed-phase liquid chromatography (RPLC), and affinity chromatography (Wang et al., 2016b). Among them, RPLC, IEC, and SEC are most commonly used for protein analysis (Mant et al., 2007). RPLC is a powerful and widely used method for the analysis of both intact and fragmented proteins. It is performed with columns containing highly pure and inert silica-based particles (usually silica particles chemically bonded with octadecylsilyl groups) as the stationary phase. In particular, for the analysis of large, intact proteins, a surface-modified, highly pure, and wide-pore size silica (e.g. ≥300 Å) is used to improve the access of proteins to the stationary phase pores (Fekete et al., 2012). In RPLC, the separation of proteins depends on the difference in adsorption coefficient or binding affinity of each analyte to immobilized stationary phase, where the hydrophobicity of the analytes determines the elution order, with the least hydrophobic molecule eluting first (Aguilar, 2004). Furthermore, the binding affinity is affected by the structure of the analytes and the nature of the immobilized ligands. In the case of IEC, the retention mechanism depends on the surface charge of the proteins and the charge of the surrounding medium; therefore, choosing an appropriate pH value is important (Kopaciewicz et al., 1983). In SEC, the separation of molecules is based on the hydrodynamic radius and the shape of the analytes rather than adsorptive interactions between the analytes and the column support (Hong et al., 2012; Kim et al., 2020, 2022b). SEC is also referred to as gel filtration chromatography (GFC) if aqueous solutions are used as the mobile phase. Since the analytes are filtered through the porous network of the stationary phase, the SEC column works like an ‘inverse sieve’ where because larger molecules cannot move through the internal pores as deeply as smaller molecules, they elute earlier (Grotefend et al., 2012). Although LC-MS/MS has become increasingly popular for protein analysis in recent years, SEC is still widely used as a powerful, more cost-effective tool.

In addition to different separation modes, HPLC offers various detection options, including UV or fluorescence detection and mass spectrometry, which may affect its sensitivity and specificity (Table 1). High recovery of proteins and peptides from the stationary phase is also important since it influence the sensitivity and reproducibility of HPLC assay (D’Atri et al., 2020). Various factors such as column dimensions, pore size, ligand type, mobile phase composition, temperature, and flow rate can greatly influence the sensitivity, specificity, and recovery in the chromatographic analysis of proteins and peptides (Josic & Kovac, 2010; Bobály et al., 2015). More details of these variables will be discussed in Section 3.2.1.

**Table 1.** Commonly used HPLC detectors (Locatelli et al., 2012; Knol et al., 2021).

Type	Features	Sensitivity
UV/VIS detector	Most common absorbance detector Easy to operate and provide good stability Wide range of wavelength selection, covering both UV and VIS ranges (typically 195–700 nm)	1 pg–1 ng
Photo diode array (PDA) detector	Detect an entire spectrum simultaneously and save time Convenient to determine the most suitable wavelength without repeating analyses Visualize the result in three dimensions (light intensity, wavelength, time)	1 pg–1 ng
Fluorescence detector	Detect fluorescent absorbance Possible to use fluorescence derivatives such as dansylchloride for some compounds having no fluorescence absorbance or low absorbance Provide high selectivity, sensitivity, and specificity Easy to operate and relatively stable	10 fg–10 pg
Mass spectroscopic detector	Technique that combines physical separation capabilities of HPLC with detection analyzer of mass spectroscopy. Provide high selectivity and sensitivity Fast detection without the need of retention time validation Useful for structure identification as well as quantitative analysis	100 ag–1 ng

#### 2.5.2. LC-MS/MS

Mass spectrometry (MS) measures the mass-to-charge ratio of ions to quantify drugs in the biological matrix. The key components of mass spectrometers include an ion source, a mass analyzer, and a detector that measures the intensity of ionized molecules using the mass-to-charge ratio (*m/z*) (Jonsson, 2001). Various ionization methods are available, including ion electron impact, chemical ionization, electrospray ionization (ESI), and laser desorption (Jonsson, 2001; Jeong et al., 2020). Among them, ESI is extensively used for quantitative protein analysis. ESI is the most effective and soft ionization technique, which produces ionized droplets without breaking chemical bonds and further fragmenting the peptides (Bolbach, 2005; Banerjee & Mazumdar, 2012). Different mass analyzers including quadrupole (Q), ion-trap (IT), time-of-flight (TOF), Fourier transform ion cyclotron resonance (FTICR), and Orbitrap are also available for the measurement of fragmented ions (Glish & Vachet, 2003). The combination of different analyzers (e.g. QTOF, triple-Q) can be used to improve the sensitivity, accuracy, and resolution of ions having similar *m/z* values in quantifying proteins or peptides (Neagu et al., 2022). However, if multiple substances having identical molecular masses exist in the sample mixture, chromatographic separation should be carried out prior to MS analysis (Karpievitch et al., 2010). Recently, a multidimensional LC strategy has been reported to improve the separation of peptides or proteins, including cation exchange and RPLC separation (CEX-RPLC) and hydrophilic interaction chromatography (HILIC)-RPLC separation (HILIC-RPLC) (Law et al., 2015; Stoll et al., 2018). In addition, Baghdady & Schug (2019a) evaluated different high pH volatile buffers and ion pairing reagents by using two different high pH resistant RPLC packing materials (silica- and polymer-based). They achieved the best chromatographic separation using triethylammonium bicarbonate at pH 10 and hybrid silica particles, suggesting that online coupling of high pH RPLC configuration to low pH RPLC for a comprehensive two-dimensional LC may be feasible for intact proteins (Baghdady & Schug, 2019a). However, multidimensional LC requires a larger amount of samples and more analysis time compared to using single LC (McDonald et al., 2002; Peng et al., 2003).

Tandem mass spectrometry (MS/MS) combines two mass analyzers in a single instrument. After a sample is ionized and mass-analyzed in the first mass analyzer, a distinct ion of interest having a particular m/z-ratio is directed into a collision cell and undergoes fragmentation by various dissociation methods, including collision-induced dissociation, higher energy collision dissociation, electron-transfer dissociation, and electron-capture dissociation (van den Broek et al., 2008). Then, the generated fragments are separated by the second mass analyzer, based on their individual *m/z* ratios. To improve the selectivity and specificity, HPLC-based separation can be performed prior to MS/MS analysis, which is known as LC-MS/MS analysis.

In current clinical practice, two representative methods for the quantification of target protein in plasma are ELISA and LC-MS/MS; the advantages and disadvantages of LC-MS/MS are summarized in comparison with ELISA in Table 3. Owing to the substantial sensitivity, selectivity, and high throughput, the LC-MS/MS assay often becomes a method of choice for the identification and quantification of proteins in complex biological samples. Selected examples for the application of LC-MS/MS in protein assays are summarized in Table 2. Accordingly, its application as an essential analytical tool is continuously expanded in the pharmaceutical R&D process. Therefore, this review will cover more details on the method development of LC-MS/MS for quantifying therapeutic proteins in biological samples such as serum and urine in the following sections.

**Table 2.** Selected examples of protein quantification using LC-MS/MS.

Drug	Biological matrix	Sample preparation	Column	Mass system	References
NVS001	Human serum	Immunoaffinity capture and protein digestion	C18 (2.7 µm) 100 × 2.1 mm	ESI-triple quadrupole	(Fu et al., 2018)
Recombinant FGF21	Human serum	Immunoaffinity capture and protein digestion	C18 (2.6 µm) 50 × 2.1 mm	ESI-triple quadrupole	(Zhao et al., 2018b)
Nivolumab	Human serum	Immunoaffinity capture and protein digestion	C18 (1.6 µm) 100 × 2.1 mm	ESI-triple quadrupole	(Millet et al., 2021)
Thyroglobulin	Chicken serum	Immunoaffinity capture and protein digestion	C18 (1.8 µm) 50 × 1.5 mm	ESI-triple quadrupole	(Shi et al., 2022)
Insulin like growth factor - I, II	Human plasma	Protein precipitation	C18 (1.6 µm) 100 × 2.1 mm	ESI-triple quadrupole	(Pratt et al., 2021)
Denosumab	Human serum	Protein digestion	C18 (2.6 µm) 150 × 3 mm	ESI-triple quadrupole	(Shida et al. 2018)
Insulin like growth factor - I	Human serum	Solid phase extraction	C18 (1.6 µm) 50 × 2.1 mm	ESI-triple quadrupole	(Tanna et al. 2020)
PEG-Interferon-α-2b	Human serum	Protein precipitation and protein digestion	C18 (3 µm) 100 × 2.1 mm	ESI-triple quadrupole	(Kusuma et al., 2021)
Tocilizumab	Human serum	Protein digestion and solid phase extraction	C18 (2.6 µm) 150 × 3 mm	ESI-triple quadrupole	(Mochizuki et al., 2022)
Parathyroid hormone-Fc	Human serum	Immunoaffinity capture and protein digestion	C18 (2 µm) 50 × 0.3 mm	ESI-triple quadrupole	(Wang et al., 2022)
Cardiac troponin I	Human plasma	Immunoaffinity capture and protein digestion	C18 (3.5 µm) 150 × 2.1 mm	ESI-triple quadrupole	(Schneck et al., 2018)
Apolipoproteins	Human serum	Protein digestion	C18 (2.7 µm) 100 × 2.1 mm	ESI-triple quadrupole	(Toth et al., 2017)
8-iso-Prostaglandin F2 alpha	Human plasma	Liquid-liquid extraction	C18 (2.6 µm) 100 × 2.1 mm	ESI-triple quadrupole	(Tomov et al., 2021)
Estrogens	Human saliva	Liquid-liquid extraction	C18 (2.5 µm) 100 × 2.1 mm	ESI-triple quadrupole	(Li et al., 2018)

**Table 3.** Advantages and disadvantages of ELISA and LC-MS/MS.

Method	Advantages	Disadvantages
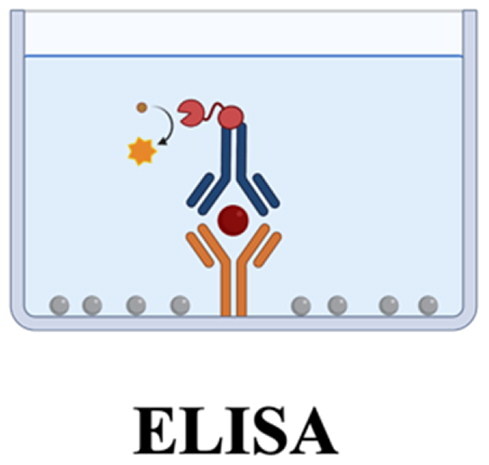	Ease-of-use Low equipment cost High throughput (microplate) High level of sensitivity	Cross-reactivity Relatively high sample volumes (100–200 µL) High cost-per-sample Low reproducibility Relatively long assay time
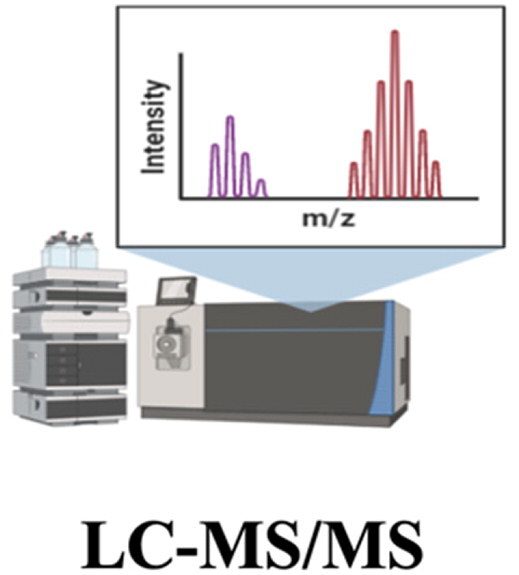	High level of selectivity and sensitivity High throughput Low sample volumes (<5 µL) Relatively low cast-per-sample High reproducibility Multiplexing	High equipment cost Complexity in the optimization of assay conditions Potential matrix effect (lowering the assay sensitivity)

## 3. Application of LC-MS/MS for quantifying proteins in plasma

### 3.1. Sample preparation

**Figure 1.**
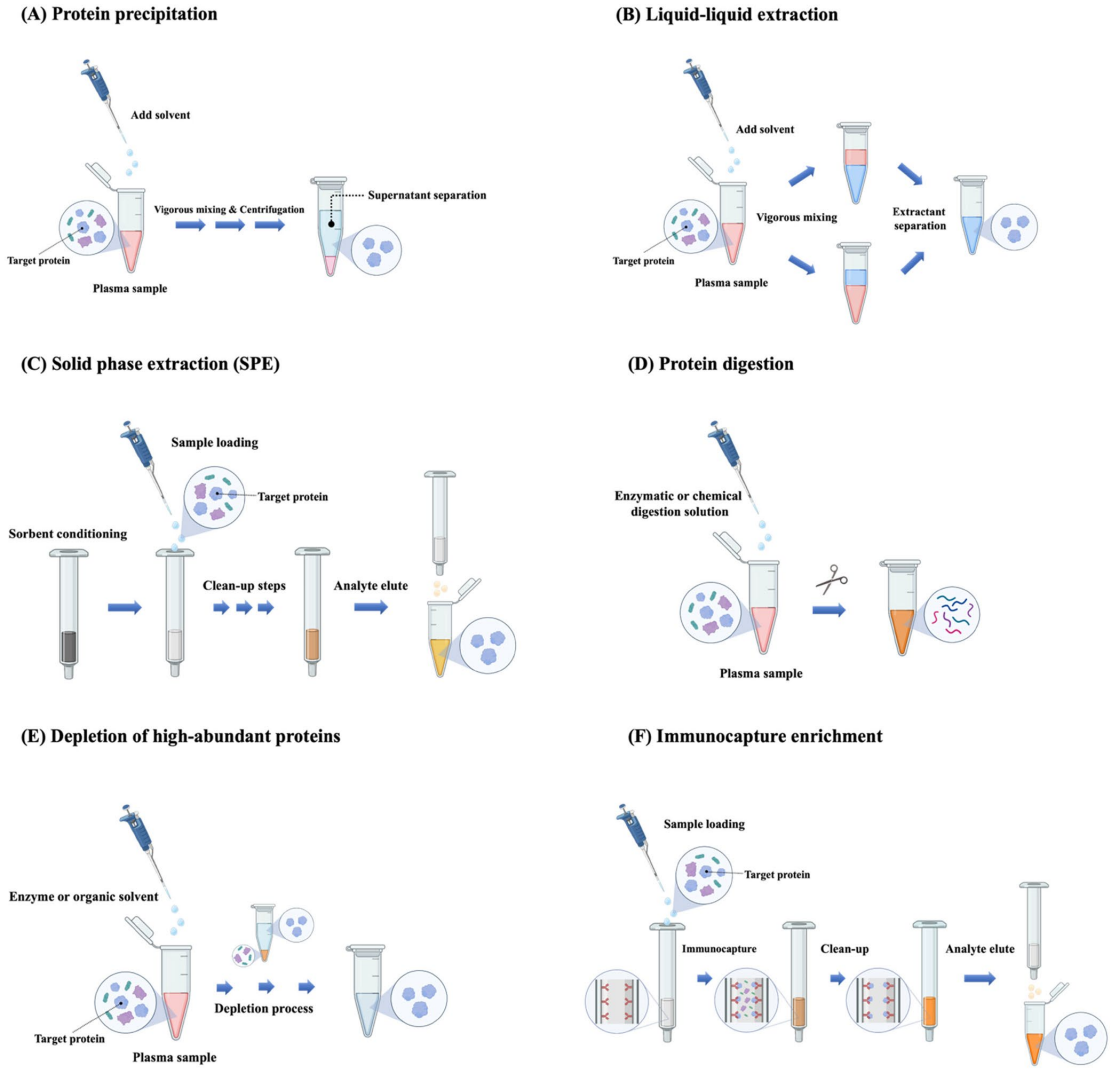
Sample preparation methods prior to the quantification of protein drugs in plasma using LC-MS/MS.

Sample preparation is a critical part of LC-MS/MS analysis for the quantitative determination of target proteins in biological fluids (Maráková, 2022). Various sample preparation methods have been developed for an efficient and reproducible LC-MS/MS analysis of protein samples. Each method has its own limitations, and no single method works for all diverse types of proteins. Furthermore, there is limited commercial availability of simple, economic, and non-immunoaffinity sample preparation options for intact proteins (Maráková, 2022). Since an efficient and robust sample preparation process allows faster and cleaner chromatographic separation prior to MS analysis, the quality of sample preparation can considerably affect the quality of data obtained from LC-MS/MS analysis. Therefore, the selection and optimization of the appropriate sample preparation method is essential to assure a highly sensitive, accurate, and reproducible LC-MS/MS analysis for protein quantitation. Some common sample preparation methods are illustrated in Figure 1 and their basic principles are briefly discussed in the following section.

#### 3.1.1. Protein precipitation (PPT)

PPT is the most widely used plasma sample preparation method for LC-MS/MS analysis because of its simplicity, fast speed, and low cost (Zheng et al., 2014a; Yuan, 2019). It is appropriate for high protein matrices such as plasma and serum, utilizing the solubility difference between protein drugs of interest and many other macromolecules in biological fluids (Thomas et al., 2021). In principle, the change in pH or hydrophobicity by adding a precipitating reagent (e.g. organic solvents, acids, salts), alters the interactions between proteins and the aqueous environment, precipitating proteins out of solution (Zheng et al., 2014a; Lim et al., 2020). In addition, binding of metals to proteins causes the denaturation and the aggregation of proteins, promoting protein precipitation (Polson et al., 2003). Generally, the protein precipitant is separated either through centrifugation or filtration, and the supernatants are used for further analysis (Yuan, 2019).

The PPT process can be automated using commercially available 96-well PPT plates that are also compatible with most LC/MS autosamplers. Generally, PPT does not require extensive method development and can be implemented with a simple generic method. In addition, PPT is very efficient, removing >90% of proteins from various animal plasma samples by using a 2:1 volume ratio of acetonitrile to plasma (Polson et al., 2003). However, it has some limitations, particularly when the plasma concentration of protein drugs is very low. If evaporation and re-constitution steps are omitted, the limit of quantitation (LOQ) of the assay may be compromised by dilution with precipitating reagents, leading to reduced assay sensitivity (Li et al., 2019). Furthermore, as various endogenous proteins or phospholipids/salts in plasma cannot be completely removed by PPT, they often interfere with the assay (Zheng et al., 2014a). PPT may also cause a loss of protein drugs during the precipitation of endogenous plasma proteins, due to protein binding or analyte stability (Zheng et al., 2014a). Thus, it is important to disrupt protein binding of the biomolecules from the biological matrices and maximize the drug recovery. Various strategies are employed to improve the drug recovery in PPT. Formic acid or ammonium hydroxide can be added into the organic solvents to reduce protein binding (Zhao & Juck, 2018). In addition, to improve the drug recovery, low volume ratios of precipitating solvents to plasma sample are preferred, generally 2:1 or even lower (Polson et al., 2003). However, it may reduce the efficiency of protein removal. To overcome these issues, PPT may be used in combination with other sample preparation techniques such as solid phase extraction or liquid-liquid extraction (Chambers et al., 2014; Miyachi et al., 2017).

#### 3.1.2. Liquid-liquid extraction (LLE)

LLE is a method that can be used to selectively extract the analyte from biological matrices using differential distribution of biomolecules into a two‐phase solution system (typically an aqueous solution and a water-immiscible organic solvent or solvent mixture) (Chang et al., 2007). In LLE, the analyte of interest in biological fluids is partitioned by adding an organic extraction solvent with vigorous mixing, followed by additional steps such as evaporation and reconstitution (Vuckovic, 2020). For improving the recovery of analytes while minimizing the matrix effect, experimental variables such as the extraction solvent, extraction buffer, pH, and the volume ratio of the sample and extraction solvent should be carefully optimized (Yuan, 2019).

In general, LLE is more efficient in removing endogenous proteins, lipids, and salts from biological samples than PPT (Yuan, 2019). LLE is simple, easy to run, and high‐throughput in a 96‐well plate format (Zheng et al., 2014b). It is also more cost-effective than solid-phase extraction (SPE). However, the application of the LLE method is mainly limited to hydrophobic proteins, as polar proteins are not efficiently extracted by typical water-immiscible organic solvents (Yuan, 2019). To overcome this issue and expand its application to hydrophilic proteins, salting‐out-assisted LLE (SALLE) has been developed as an alternative LLE (Tang & Weng, 2013). It employs the salting-out effect of a water-miscible organic solvent by the addition of a substance inducing phase separation from an aqueous solution, achieving the simultaneous extraction of target compounds into a separated organic solvent phase (Zhang & Xiong, 2019). As salts and extraction solvents have a great influence on the extraction efficiency and drug recovery, their selection should be carefully optimized (Zhang & Xiong, 2019). The most commonly used organic solvents for SALLE are acetonitrile, acetone, and isopropanol, while most commonly used salting-out agents include sodium chloride, calcium chloride, magnesium sulfate, and ammonium sulfate (Tang & Weng, 2013; Zhang & Xiong, 2019).

Although SALLE offers an alternative for the extraction of hydrophilic compounds that are not efficiently extracted by conventional LLE, it is not limited to only polar drugs. Compared to PPT, SALLE is similarly simple but provides cleaner extracts due to the phase separation. It is also faster and more cost-effective than conventional LLE. In most cases, SALLE does not require evaporation and reconstitution steps, allowing subsequent LC-MS/MS analysis directly in the organic phase or after a simple dilution (Zhang & Xiong, 2019). Particularly, SALLE in 96-well automation can be easily integrated into the high-throughput LC-MS/MS assay to increase productivity.

#### 3.1.3. Solid-phase extraction (SPE)

SPE is an efficient method providing more selective extraction of various compounds via chromatographic separation using cartridges packed with silica-based or polymer-based sorbents (Badawy et al., 2022). The selection of an appropriate SPE sorbent is critical for the successful extraction of target analytes and should be done by taking into account various physicochemical properties (e.g. polarity, basicity, charge) of analytes. Similar to HPLC columns, many stationary phase options are available such as reversed phase, ion exchange, normal phase, and mixed mode phases (Sentellas et al., 2020). Among them, the most commonly used SPE materials for the isolation of proteins and peptides are reversed-phase and ion-exchange materials. Dual retention modes combining different types of SPE are often used to improve the selectivity and sensitivity, particularly prior to the quantitative analysis of therapeutic proteins (Yang et al., 2007). For example, RP-SCX-SPE, which combines reversed phase-SPE (RP-SPE) with strong cation exchange-SPE (SCX-SPE), could remove the background peptides and other interfering substances in a biological matrix more efficiently than RP-SPE alone, thereby greatly reducing the background noise and improving the assay sensitivity of LC-MS/MS (Yang et al., 2007). SPE materials are also available in various formats including cartridges, plates, micropipette tips, and magnetic beads (Bladergroen & van der Burgt, 2015).

Although SPE is a commonly used sample preparation method, it has issues including poor recovery and reproducibility, incomplete sample cleanup, and high matrix effect (Płotka-Wasylka et al., 2015; Badawy et al., 2022). To overcome these drawbacks, new microextraction techniques have been developed, including solid-phase microextraction (SPME), dispersive micro-SPE (DMSPE), and magnetic SPE (MSPE) (Chen & Bartlett, 2012). For example, micro-elution SPE is a miniaturized solid-phase extraction method, enabling minimal elution volumes to deliver concentrated samples for high-sensitivity analysis. In a conventional SPE plate or column, higher eluting solvent volumes may require an evaporation step to concentrate samples. However, micro-elution SPE containing a sorbent bed as little as 2 mg, requires typical sample volumes of 10–100 µL and elution volumes as low as 25 µL (usually <150 µL), diluting samples without losing sensitivity (Maráková et al., 2023). Micro-elution SPE technique also offers shorter operation time and lower cost. Moreover, it is suitable for hydrophobic proteins and the direct analysis of analyte without drying and reconstitution step (Maráková et al., 2023).

Unlike HPLC, SPE is typically a low resolution and very low-pressure method that helps remove interfering substances or concentrate a sample, thereby improving assay sensitivity. SPE provides much cleaner extracts than PPT and uses significantly smaller volumes of solvent than LLE (Yuan, 2019). It can reduce harmful compounds introduced into the LC system, thereby extending the life-time of analytical columns and instruments. Furthermore, SPE can be automated in a 96‐well plate format that are directly coupled to LC-MS/MS analysis (Sentellas et al., 2020). As a result, SPE is widely used in pharmaceutical applications. However, compared to PPT and LLE, its cost is higher and the optimization of its experimental variables takes longer time (Zheng et al., 2014a).

#### 3.1.4. Protein digestion

Compared to proteins, peptides are easier to fractionate by LC and are ionized and fragmented more efficiently. Thus, in the quantitative analysis of proteins in plasma or other biological fluids, protein digestion is actively employed to convert protein analytes to peptides mixture and then to quantify one or more of peptides as a surrogate by LC-MS/MS. Protein digestion can be done enzymatically or non-enzymatically (Angel et al., 2012). The enzymatic or chemical digestion of proteins cleaves the protein into smaller peptides having higher chemical tractability, lower molecular mass, and fewer charges, thus leading to more efficient chromatographic separation and better detection by the mass analyzer (Compton et al., 2011; Willems et al., 2021). The most widely used digestion methods employ sequence-specific proteases such as trypsin, chymotrypsin, endoproteinase Glu-C, and Lysyl-endopeptidase (Switzar et al., 2013). Among proteolytic enzymes, trypsin is a gold standard owing to its high specificity and low cost, cutting at the carboxyl side of arginine (Arg) and lysine (Lys) residues. It produces positively charged peptides with an average size of 700–1,500 Da, which are easily detectable by MS (Burkhart et al., 2012). Selection to perform in-solution or in-gel digestion depends on the sample amount and/or its complexity. In-solution digestion is generally advantageous for small sample volumes with low to moderate complexity and is more amenable to high throughput. In in-solution digestion, proteins typically undergo irreversible cleavage of disulfide bonds via reduction and alkylation, prior to digestion, to allow immediate access of trypsin to internal cleavage sites and achieve high protein sequence coverage during MS analysis (Medzihradszky, 2005). However, reduction and alkylation steps can be excluded if high sequence coverage is not required. Although trypsin is highly active and tolerant of many additives, tightly folded proteins can be resistant to trypsin digestion. Post-translational modifications also present a challenge for trypsin-based digestion since glycans can limit trypsin access to cleavage sites, and acetylation makes Lys and Arg residues resistant to trypsin digestion (Saveliev et al., 2007). In addition, if the distribution of tryptic cleavage sites is suboptimal, the obtained peptides may be too long or too short for MS analysis. To overcome these issues, various alternative proteases, including Glu-C, Lys-N, Lys-C, Asp-N, and chymotrypsin are commercially available, which complement trypsin and allow more efficient protein analysis with MS. Extraction of the protein prior to enzyme digestion and/or extraction of the surrogate peptide after enzyme digestion can also be employed to improve the selectivity and sensitivity of protein analysis (Li et al., 2011).

As an alternative to enzymatic digestion, nonenzymatic digestion of proteins can be performed via chemical cleavage by dilute acid solutions (e.g. formic acid, hydrochloric acid, acetic acid), cyanogen bromide, 2-nitro-5-thiocyanobenzoate, and hydroxylamine (Crimmins et al., 2000; Switzar et al., 2013). In recent years, electrochemical oxidation-based nonenzymatic cleavage of proteins has been reported to allow specific cleavage at Tyr and Trp, exhibiting the advantages of rapid reaction and online coupling to an LC-MS system (Permentier et al., 2003). To enhance the digestion of diverse proteins, several approaches have also been explored, including the combined use of chemical and enzymatic methods (Choudhary et al., 2003) and multiple enzyme digestion strategy using the combination of enzymes having different specificity (Swaney et al., 2010). Similarly, immobilization of enzymes onto a solid surface can be used to increase enzyme stability and digestion efficiency (Massolini & Calleri, 2005; Yamaguchi et al., 2010) . In addition, various approaches such as elevated temperature, microwave, ultrasound, and high pressure have been used to accelerate digestion (Switzar et al., 2013). The physicochemical properties of protein samples are critical to determine how they are digested and prepared further for LC analysis. Filtration is often applied to samples containing particles such as undissolved salts. The experimental conditions for each method should be tailored to specific samples and MS techniques for optimal and reproducible results.

#### 3.1.5. Depletion of high-abundance proteins

High-abundance proteins (HAPs) present in serum, plasma, and other physiological fluids can hinder the identification and characterization of important low-abundance proteins (LAPs) by limiting the dynamic range for mass spectral analyses due to the masking effects of HAPs (Pietrowska et al., 2019). Therefore, depletion of HAPs in biological samples has become a routine strategy for enhancing the detection sensitivity of LC-MS/MS analysis.

High abundant plasma proteins include albumin, IgG, antitrypsin, IgA, transferrin, haptoglobin, and fibrinogen (Millioni et al., 2011; Pietrowska et al., 2019). Depletion of these HAPs in biological samples has become a routine strategy for enhancing the detection sensitivity of LC-MS/MS analysis. Depletion of HAPs improves the dynamic range for LC-MS/MS analysis and the loading capacity, resulting in the simplification of a complex system and enabling the detection of low-level target proteins. Several fractionation methods are available for protein depletion, which are based on the separation of proteins by physicochemical properties (charge or size) or biochemical properties (immunoaffinity) (Ly & Wasinger, 2011). In particular, immuno-based depletion methods involve the selective binding of target proteins to the stationary phase using immobilized antibodies or other molecules with high affinity and specificity for the targets (Filip et al., 2015). As a result, they have high specificity and efficiency, achieving rapid purification or concentration of the analytes.

An alternative strategy to isolate LAPs is based on an immobilized, combinatorial peptide ligand library (Righetti & Boschetti, 2015; Lee et al., 2019). Highly diverse hexapeptides are bound to a chromatographic support where each unique hexapeptide binds to a unique protein recognition site. In this approach, HAPs saturate their ligands and excess proteins are washed out during the process while LAPs are concentrated on their specific ligands. Several protein depletion kits are commercially available, simultaneously depleting multiple HAPs (approximately 7–20 HAPs).

Depletion of HAPs by immunoaffinity columns may be followed by denaturation, reduction, desalting, and tryptic digestion of low-abundance target proteins to improve the accuracy and the precision of the assay (Shi et al., 2012b). Such an integrated sample preparation system can be further integrated with LC-MS/MS to achieve high-throughput protein quantification in biological samples.

#### 3.1.6. Immunocapture enrichment

Immunocapture enrichment is widely used to isolate and enrich the target proteins or peptides from the biological matrices. It employs capture reagents (usually antibodies) for the specific and high affinity interaction with the target analytes, offering a highly selective sample cleanup and enrichment procedure (Zhao et al., 2018a). In addition, combination of immunocapture enrichment with LC-MS/MS analysis provides a viable option for the quantitative analysis of LAPs or signature peptides in biological matrices. Capture reagents are usually immobilized on a solid support. During the immunocapture process, the target analyte binds specifically and tightly to the capture reagent on a solid support but other matrix components that do not bind to the capture reagent are removed from the solid support by washing with a buffer (Zhao et al., 2018a). Then, the target analytes are eluted from the capture reagents by adding acids, followed by LC-MS/MS analysis. The immunocapture process produces a clean, enriched extract and greatly reduces matrix effect in LC-MS/MS analysis, thereby improving assay sensitivity. There are several critical factors in developing a reliable and robust immunocapture-LC-MS/MS assay, including reagent selection and designing of capture format. Particularly, capture reagents are the most crucial component in the immunocapture-LC-MS/MS analysis, since their quality directly affect the assay specificity, reproducibility, sensitivity, and robustness (Zhao et al., 2018a). Capture reagents are categorized into two groups; generic capture reagents and specific capture reagents. Generic capture reagents bind to various common regions (e.g. Fab or Fc) of proteins but have different binding affinities across protein species (Fung et al., 2016). Since the low specificity issues of generic capture reagents can be overcome by selective mass detection, generic capture reagents are useful in a wide range of applications. On the other hand, analyte-specific capture reagents (e.g. antipayload, antitarget, antipeptide antibodies) are required to improve the specificity of immunocapture, providing a more reliable and robust assay (Zhao et al., 2018a).

Instead of proteins enrichment with antiprotein antibodies, an alternative approach involves immunocapturing a surrogate peptide of protein drugs using anti-peptide antibodies after enzymatic protein digestion (Anderson et al., 2004). This approach is commonly called, ‘Stable Isotope Standards and Capture by Anti-Peptide Antibodies (SISCAPA)’ (van den Broek et al., 2015). After adding a stable isotope-labeled surrogate peptide as an internal standard into digested samples, both the labeled and nonlabelled surrogate peptides are captured by a sequence-specific antipeptide antibody, then enriched and separated from biological matrices for LC-MS/MS analysis (Anderson, 2004). Compared to antiprotein immunocapture-LC-MS/MS assays, antipeptide immunocapture-LC-MS/MS assays provide higher sensitivity particularly for LAPs in plasma by minimizing potential interferences (Becker & Hoofnagle, 2012). Considering the labor-extensive immunocapture steps, automated immunocapture platforms are desirable to improve the assay throughput. Several immunocapture platforms are commercially available, including bead-based, cartridge/tip-based, and plate-based platforms (Neubert et al., 2020). Among them, magnetic bead-based platforms are commonly used due to their commercial availability, cost effectiveness, and application flexibility. The selection of platforms suitable for the assay may depend on the sensitivity requirement and availability of reagents.

### 3.2. Optimization of LC-MS/MS conditions

**Figure 2.**
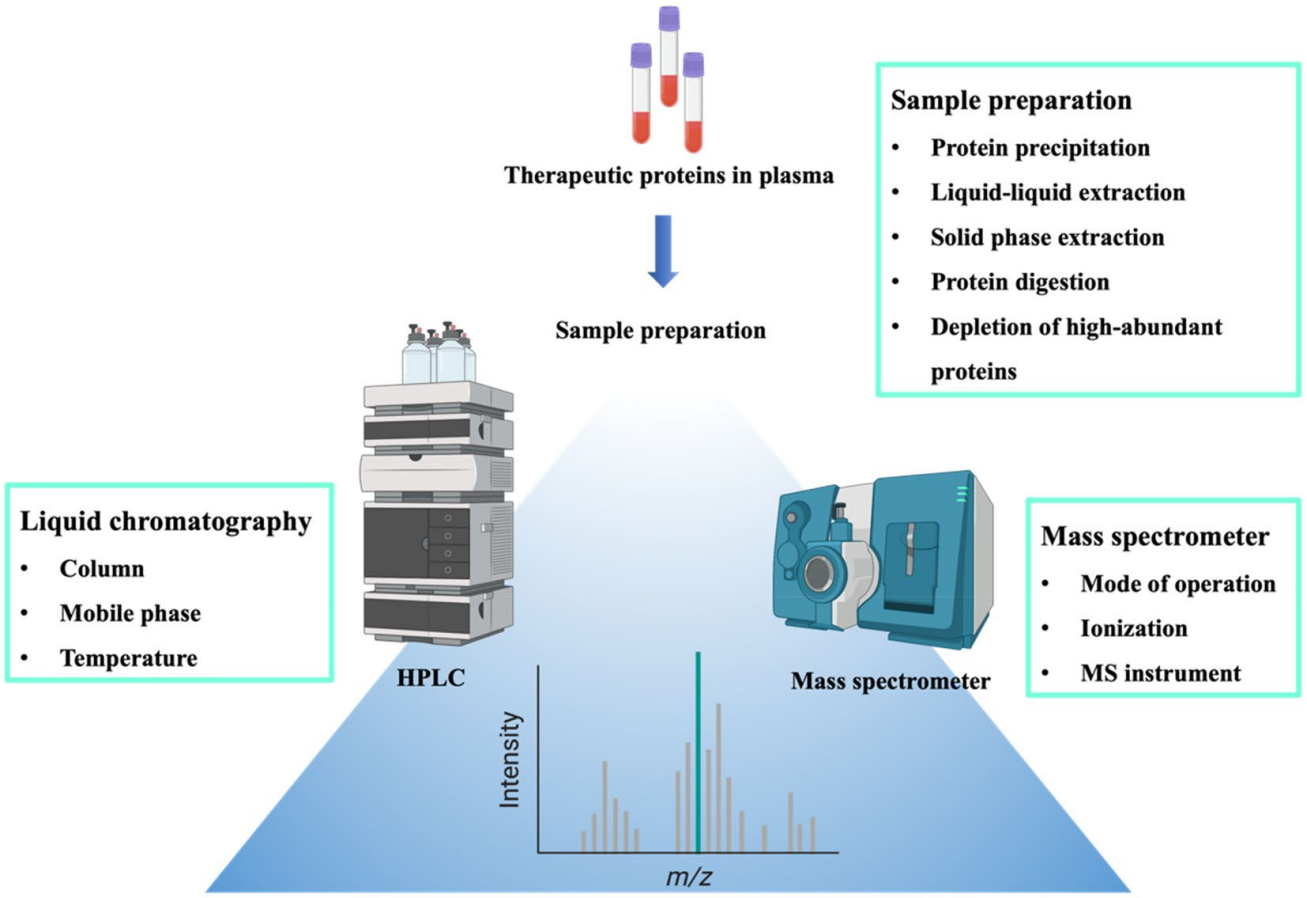
Workflow and critical factors in LC-MS/MS analysis of protein samples.

Reversed-phase HPLC (RP-HPLC) plays a central role in the quantitative analysis of protein-based drugs due to its versatility, sensitivity, and compatibility with mass spectrometry. In addition, RP-HPLC has high resolution capability to separate structurally similar proteins and peptides, facilitating its widespread use for protein analysis. Therefore, combination of RP-HPLC with highly selective and sensitive tandem MS allows high-throughput assay for the identification and the quantitation of low-abundance target proteins in complex biological samples (Donato et al., 2012). Various parameters in a workflow should be optimized for efficient LC-MS/MS analysis (Figure 2). Prior to mass analysis, the chromatographic separation should be optimized to provide high resolution, capacity, and fast turnaround time. In addition, various factors affecting MS detection should be optimized to produce highly accurate and reproducible results (Nehete et al., 2013). Some critical factors affecting the chromatographic separation and MS detection are briefly discussed in the following section.

#### 3.2.1. Liquid chromatography

##### 3.2.1.1. Column

In RP-HPLC, proteins or peptides are adsorbed on the hydrophobic surface of the stationary phase (Neverova & Van Eyk, 2005; D’Atri et al., 2020). Owing to the large size of proteins, only a portion of the protein adsorbs to the hydrophobic surface of stationary phase and the greater part of the protein lies above the hydrophobic surface in contact with the mobile phase. When a specific concentration of organic solvent is reached, the protein desorbs from the surface and elutes from the column (Carr, 2016). Given that the separation of proteins via this adsorption and desorption process depends on the extent of interaction with particles packed in a column, the surface characteristics of the packing materials greatly affects the chromatographic separation of the protein samples (Issaq et al., 2003; Wang et al., 2016a). In RP-HPLC, silica particles are widely used as the stationary phase due to their physical robustness, stability, and diversity in pore size and particle diameter (Borges, 2015). Various functional groups are introduced to modify the silica surface to achieve different degrees of hydrophobicity (Corran, 1989). Among them, an octadecylsilyl (ODS) column filled with silica particles chemically bonded with ODS groups is most widely used in RP-HPLC. It is commonly used for the separation of peptides (typically less than 2,000–3,000 Da) after protein digestion. Similarly, silica surfaces with less hydrophobic attachment, including butyl (C4) or phenyl phases, are suitable for the separation of large or hydrophobic peptides (Zhou et al., 1991).

The shape, pore size, and diameter of silica particles also affect the resolution in HPLC assays of complex biological mixtures containing proteins. While resolution can be improved by decreasing the particle diameter, the most commonly used particle diameters for analytical RP-HPLC are in the range of 3–5 µm (Aguilar, 2004). The pore size of packing materials is also an important factor to enhance the resolution. Generally, a silica column packed with porous materials having a pore size of ∼ 100 Å results in poor protein separation, since proteins cannot enter small pores (Kirkland et al., 2002). Only after protein digestion, the obtained small peptides can enter the small pores of the silica packing materials and interact with the hydrophobic surface. Therefore, silica columns having porous materials (pore size ≥300 Å) are commonly used for the separation of proteins with better resolution, since the diameter of solute molecules should be at least one-tenth the size of the pore diameter to avoid restricted diffusion of the solute and make the total surface area of the sorbent material accessible (Wei et al., 1997; Carr, 2016). Particles with 6,000–8,000 Å pores with a network of smaller pores of 500–1,000 Å have achieved rapid protein separations (Paliwal et al., 1996). In addition, the purity of the silica particles affects peak separation. Silica particles having metal ion impurities cause peak tailing and poor resolution (Zhang et al., 2014). Similarly, size (volume) and pH of the samples should also be considered in column selection, since they may determine the pore and particle size as well as the types of bonded phases in the column packing materials (Kromidas, 2008; Žuvela et al., 2019; Baghdady & Schug, 2019b). However, the column length does not seem to be critical in the separation of protein samples. Once proteins are desorbed near the top of the column, the interaction of proteins with the hydrophobic stationary phase is minimal as proteins move down the column (Carr, 2002; Fekete et al., 2021). ; Recently, Fekete et al. (2021) reported the utility of ultrashort columns for the high-throughput RPLC analysis of therapeutic proteins. They observed that ultrashort columns of only 5 mm length could separate antibody fragments and antibody-drug conjugates in less than 30 s, providing acceptable performance compared to regular columns (100–150 mm length). This short column has advantages including significantly shorten analysis time and less protein hydrolysis due to lower residence times.

##### 3.2.1.2. Mobile phase

The selectivity and resolution of HPLC can be improved by tuning the mobile phase in terms of composition, elution mode, and flow rate (Aguilar, 2004). The mobile phase should make use of the highest purity water, solvents, and buffers to lower the background noise, since limits of detection and quantitation in LC-MS/MS analysis will be compromised by a high level of background noise. Based on eluent strength, viscosity, and polarity, organic solvents such as acetonitrile, methanol, and 2-propanol are commonly used for protein sample analysis (Corran, 1989). In addition to eluotropic effects, the organic solvent also influences the conformational change of proteins, providing an additional effect on the selectivity and the recovery of proteins (Aguilar, 2004). The mobile phase also contains ion-pair reagents such as trifluroacetic acid (TFA), phosphoric acid, and heptafluorobutyric acid, improving the resolution (McCalley, 2005; Dong, 2006). Given that TFA significantly decreases the MS sensitivity due to ion suppression effects during the electrospray process via the formation of ion pairs with analytes, the use of TFA should be minimized in LC-MS/MS analysis or replaced by an alternative (Mallet et al., 2004; Maráková et al., 2020). As an alternative acidic mobile phase additive, difluoroacetic acid (DFA) is promising for the analysis of peptides and proteins, effectively lowering pH to suppress deleterious silanol interactions. While it is a strong ion-pairing agent suitable to improve RPLC separations, it does not decrease MS sensitivity as much as TFA (Maráková et al., 2020).

The mode of elution also has a significant impact on the resolution of protein samples. The stoichiometric displacement model (SDM) is an equilibrium model first proposed for RPLC, in which retention of proteins is a function of the number of solvent molecules required to displace a protein from the stationary phase ((Xindu & Regnier, 1984). When the solvating power of mobile phase is strong enough to break the multiple interaction between proteins and the stationary phase, proteins are desorbed and eluted from the stationary phase (Kopaciewicz et al., 1983; Xindu & Regnier, 1984; Regnier, 1987). In general, gradient elution provides higher resolution in the chromatographic separation of protein mixture than isocratic elution. The retention of proteins changes abruptly when the organic solvent reaches the concentration for desorption, providing sharp peaks of the protein analytes (Carr, 2016). Due to the large change in retention with small changes in organic solvent concentration, isocratic elution is seldom used with protein samples. In particular, the adjustment of the gradient slope is essential for optimizing the resolution of protein samples where a lower gradient slope or a slower rate of change in organic solvent concentrations provides better peak separation (Aguilar, 2004). Moreover, the selection and optimization of the gradient conditions primarily depends on the physicochemical characteristics of the target protein analyte. The separation of protein mixtures can be improved by longer gradient elution by increasing the residence time and mass transfer of the solute onto the stationary surface, but it may also accentuate the degree of denaturation (Kumpalume & Ghose, 2003; Burdette & Marcus, 2013).

The flow rate should also be optimized to achieve rapid separation and analysis of proteins. In contrast to small molecules, large biomolecules having low diffusivity constants show band broadening with increasing flow rates (mass transfer effect) (Knox & Scott, 1983; Ghosh et al., 2013; Podgornik et al., 2013). At high flow rates, these large molecules cannot travel in and out of porous silica particles as fast as mobile phase elution rate, leading to insufficient time for interacting with the stationary phase and therefore, to broad peaks. Hence, it is necessary to optimize the flow rate of the mobile phase to avoid potential band broadening (Martin & Guiochon, 2005).

##### 3.2.1.3. Temperature

Temperature plays an important role in optimizing the chromatographic separation of protein samples since it has a profound effect on the viscosity of the mobile phase and the diffusion of the analytes (target proteins). In general, the lower viscosity of the mobile phase and greater solute diffusivity at elevated temperatures result in more symmetrical and narrower peaks, improving the resolution (Zhu et al., 2004). In addition, the reduced viscosity of the mobile phase at elevated temperatures decreases the running time and achieves good efficiency at higher flow rates compared to those at ambient temperature, thereby increasing the speed of assay (Issaq et al., 2003). The conformational structure and the binding of proteins on the hydrophobic surface of the stationary phase can also be manipulated by changing the temperature, thus improving assay selectivity (Lazoura et al., 1997). Overall, temperature affects the chromatographic elution profiles of protein samples, whereby elevated temperatures may improve the selectivity and recovery of large or hydrophobic proteins. However, there should be caution that high temperature can affect the protein stability and cause the conformational change or thermal degradation of proteins (Staub et al., 2011).

##### 3.2.1.4. Internal standards

LC-MS/MS analysis has an inherent uncertainty from each process including extraction, pre-analytical sample treatments, chromatography, and mass detection. To minimize the variations resulting from sample preparation and analytical procedures, structurally related compounds have been used as internal standards (IS). In general, IS are added to the sample at the beginning of the sample preparation and go through the entire process with the target analytes. However, these conventional IS are chemically different from the analytes, thus behaving differently. Therefore, the analytical uncertainty is mitigated by using conventional IS but not fully compensated.

Given that isotopic analogues of the analytes are chemically identical to the analytes but have different mass, isotope-labeled IS can mirror the analytes at each stage of the process while they can be distinguished from the analyte by the mass detection (Ciccimaro & Blair, 2010). Accordingly, stable isotope labeled (SIL) standards are employed in quantitative LC-MS/MS analysis, where some atoms of the analyte are replaced by their stable (non-radioactive) isotopes such as deuterium (^2^H or D), ^13 ^C or ^15 ^N. As with the conventional IS, SIL standards should be added to the sample at the beginning of the procedure. Since SIL standards are uniquely matched to their corresponding analytes, an individual SIL standard is required for the quantification of each analyte in a multi-analytes LC-MS/MS (Zhou et al., 2017). Several critical factors should be considered for the selection and use of a SIL standard for LC/MS-MS analysis, including stability of the label, optimal mass difference between the analyte and IS, isotopic purity, and molecular sites of the isotopic labels (Nasiri et al., 2021). For example, chemical exchange of deuterium with protons from solvent or matrix components results in the loss of labeled IS, and thus isotope labeling should be positioned on non-exchangeable sites. In addition, if a molecular fragment is detected in MS analysis, the isotope labels should be positioned on the fragment of interest. A mass difference between the analyte and the SIL standard should be also suitable for avoiding the overlap of spectral lines (Jenkins et al., 2015). Isotopic purity is also important. SIL standard should be free of unlabeled species, where unlabeled molecule is undetectable or at a level not to cause interference.

In recent years, SIL analogues of target proteins are widely accepted as the optimal IS for accurate quantification of proteins using LC-MS/MS analysis (Faria & Halquist, 2018). Since a SIL protein exhibits the similar physiochemical behavior to the target analyte, the addition of a SIL-protein IS at the beginning of the sample preparation can control the variations in extraction, enzymatic digestion, LC, and MS detection, thereby increasing the accuracy and precision of the quantitative assay. Regulatory guideline also recommends the use of a SIL-protein IS in quantitative LC-MS/MS analysis (Kaza et al., 2019).

#### 3.2.2. Mass spectrometry (MS)

##### 3.2.2.1. Mode of operation

The multiple reaction monitoring (MRM) technique using a triple quadrupole (Q) mass spectrometer is the most popular for the quantitative analysis of protein drugs in complex biological matrices. In a triple Q mass spectrometer using the MRM mode, a precursor ion of interest is first selected in the first mass analyzer (Q1) based on its accurate mass (Pan et al., 2009). Then, the pre-selected precursor ion is transmitted and fragmented in a collision cell, producing a range of daughter ions. One (or more) of these resulting daughter ions are selected and mass analyzed in a second mass analyzer (Q3). In the MRM mode, monitoring more than one MS-MS transition for each target species allows the detection of multiple components in a single LC-MS/MS run (Cohen Freue & Borchers, 2012). As a result, multiple proteins can also be quantified in a single run, depending on the MS instrument, the numbers of transitions monitored for each peptide, and the number of peptides monitored for each protein (Pan et al., 2009).

To establish the optimal MRM condition for high accuracy and specificity, there are some important factors to be considered. Protein quantification is often based on the digestion of proteins of interest and subsequently measuring one or more surrogate peptides. In this case, the selection of the appropriate signature peptide as a surrogate analyte is important (Rauh, 2012). Peptides under electrospray conditions may present various ionized patterns depending on their size and structure. Based on MS responses from ionized peptides, a signature peptide with the highest MS response is selected and the transition-dependent MRM conditions such as source voltages and collision energy should be optimized (Ewles & Goodwin, 2011). The selected signature peptide should have high specificity and be efficiently ionized. Furthermore, it should be a suitable surrogate for the target protein of interest. The preferred length of signature proteins is ∼10–20 amino acid residues (Rauh, 2012). It is recommended to monitor at least three peptides for each target protein to enhance assay specificity and decrease interference from other plasma proteins (Unwin et al., 2005; Rauh, 2012). Although digestion-based methods are successful and widely utilized, it should also be noted that a surrogate peptide may represent only a small percentage of the total protein (van de Merbel, 2019). Consequently, quantification of intact proteins is increasingly being used in LC-MS/MS assay.

In addition to MRM, parallel reaction monitoring (PRM) mode has been reported in recent years, which provides high resolution and full scan MS/MS data (Bourmaud et al., 2016; Zhou et al., 2016; Kisiala et al., 2019). It allows for the highly specific extraction of signals for target peptides of interest, thereby restricting the interference from isobaric contaminants (Shi et al., 2012a; Liebler & Zimmerman, 2013).

##### 3.2.2.2. Ionization

Ionization is a critical step in MS-based protein analysis. Various ionization techniques are available, electrospray ionization (ESI), atmospheric pressure chemical ionization (APCI) and matrix-assisted laser distortion ionization (MALDI) (Awad et al., 2015; Sedláčková et al., 2022). Among them, ESI has been widely used for protein analysis. It is performed in solution, where (i) the sample is sprayed into the MS, (ii) as the droplets evaporate, electrical charges are transferred to molecules present in the droplet and multiple, charged ions are produced, and (iii) the mass analyzers generate complex ESI spectra (Kebarle & Verkerk, 2009). The flow rate of ESI is an important factor for improving the ionization of analytes and the relatively fast flow rates of conventional ESI (>100 µL/min) can limit the effective ionization of target analytes. To overcome this issue, nano-ESI devices have been developed, operating at very low flow rates (10–100 nL/min) and reducing ionization suppression effects (Schmidt et al., 2003; Tang et al., 2004). Furthermore, advancement in ionization devices using single- or dual-stage ion guides, ion funnels, and ion funnel trap devices improves ion transmission in the atmospheric pressure/vacuum interface (Kelly et al., 2010; Belov et al., 2011; Hossain et al., 2011).

##### 3.2.2.3. MS instrument

A triple quadrupole mass spectrometer provides a wide dynamic range (>10^5^), high sensitivity, and low measurement variation in MRM (Liebler & Zimmerman, 2013). However, it results in a relatively low resolution of precursor *m/z* measurements, which may be due to interference from nominally isobaric background contaminants in complex mixtures (Liebler & Zimmerman, 2013). In addition to a triple quadrupole mass spectrometer, the quadrupole-time-of-flight (QqTOF) instrument can be used for quantifying proteins. While a mass-resolving quadrupole (Q1) and a collision cell are similar to a triple quadrupole, the third quadrupole (Q3) is replaced by a TOF mass analyzer, improving the sensitivity, accuracy, and mass resolution for both precursor and product ion spectra (Steen et al., 2001). In particular, Q-TOF-based, high-resolution mass spectrometry (HRMS) is a promising approach to improve accuracy and mass resolution (van Dongen & Niessen, 2012). The main advantage of HRMS is its ability to separately detect the responses of molecules having very close molecular masses, thereby providing a more selective detection of a surrogate peptide. Since a Q-TOF-based HRMS system narrows the mass extraction window to a much lower extent than a triple quadrupole, most of the ions of a surrogate peptide can be detected while a major part of the interference from digested plasma proteins is no longer selected for detection (van de Merbel, 2019). It can also measure highly charged intact proteins (Furlong et al., 2012). In recent years, HRMS using Orbitrap mass analyzer is available as another powerful tool for quantitative analysis of proteins and peptides. Orbitrap mass analyzer consists of a spindle-like central electrode and two outer electrodes, trapping of ions in electrostatic fields (Zubarev & Makarov, 2013). With voltage applied between the central and outer electrodes, injected ions oscillate harmonically along the central electrode and the differential image-current is detected by the outer electrodes, Fourier-transformed into the frequency domain, and then converted into a mass spectrum (Zubarev & Makarov, 2013). The resolution is dependent on the number of harmonic oscillations detected. Similarly to Q-TOF, most of Orbitrap analyzers are employed in hybrid (combining a quadrupole analyzer with Orbitrap (Q-Orbitrap)) rather than stand-alone configuration. With continuous technical improvement, Orbitrap-based HRMS becomes more powerful analytical platform, providing high resolution, mass accuracy, and excellent sensitivity in various analytical application.

The inclusion of an additional separation step after chromatographic retention or mass selection can also improve the selectivity of MS detection (van den Broek et al., 2013). Various approaches, including high field asymmetric waveform ion mobility spectrometry (FAIMS) and differential mobility spectrometry (DMS) have been applied for ion mobility differentiation, reducing interference in the LC-MS/MS analysis of proteins (Kanu et al., 2008; Xia et al., 2008; Klaassen et al., 2009). However, FAIMS and DMS have relatively long residence times and may result in broader and lower peaks due to potential loss of analytes (Xia et al., 2008).

## 4. Summary and future perspectives

Complex biological matrices such as plasma and urine cause many difficulties in developing efficient quantitative assays for protein drugs. This review covers various approaches to overcome multiple issues in protein assays, with emphasis on sample preparation and quantitation by LC-MS/MS. Although various analytical methods are available for quantifying protein drugs in biofluids, there is no ideal or universal method that is applicable to a diverse range of therapeutic proteins or to all circumstances. Each method has its own advantages and disadvantages. In general, the choice of method is based on the compatibility of the analytical method with the analyte of interest and on potential interfering substances included in the samples. It is also important to select a method that requires the least manipulation or pretreatment of the samples to reduce the interference from co-existing substances. Additional criteria for the selection of method include assay range, required sample volume, turnaround time, and throughput.

In recent years, LC-MS/MS analysis has become the method of choice for the detection, identification, and quantitation of proteins in complex biological matrices. The accuracy, sensitivity, and flexibility of MS instruments facilitate the wide application of LC-MS/MS in the pharmaceutical development of protein drugs. Given that the quality and reproducibility of sample preparation significantly affect MS detection, proper sample preparation is a critical step in LC-MS/MS. Clean samples with limited sample complexity can minimize the suppression of ionization by high-abundance species, improving the selectivity and mass resolution. Furthermore, protein assays tend to move from indirect quantification of a surrogate peptide after protein digestion to direct analysis of the intact protein analyte, promoting the development of materials with improved separation properties for proteins. Therefore, sample preparation, chromatographic separation, and mass detection should be properly integrated into robust workflows for LC-MS/MS analysis. In recent years, instruments have become highly advanced, automated, and equipped with high-end information technology, allowing high-throughput, automated sample analysis, data processing, and storage. User-friendly software is also desirable for robust deconvolution of highly complex mass spectra. Continuous advancement in analytical instruments, devices, and software with other complementary technologies will further improve the bioanalytical performance, complete system integration, and automation, while reducing the workload.
